# 71 Underweight Adult Burn Survivors Report Significantly Worse Physical Function and Social Participation

**DOI:** 10.1093/jbcr/iraf019.071

**Published:** 2025-04-01

**Authors:** Deborah Choe, Ayumi Saito, Andrew Humbert, Karen Kowalske, Jamie Oh, Kristine Winnek, Jeffrey Schneider, Colleen Ryan, Haig Yenikomshian

**Affiliations:** University of California Keck School of Medicine; University of Washington; University of Washington; University of Texas Southwestern; Medstar Washington Hospital Center; Los Angeles General Medical Center; Spaulding Rehabilitation Hospital, Harvard Medical School; Shriners Children’s Boston and Massachusetts General Hospital; University of California Keck School of Medicine

## Abstract

**Introduction:**

Burns can cause serious physical injury and psychosocial distress. Extremes of body weight can also result in impaired physical recovery and emotional distress. However, the impact of BMI on long-term self-reported outcomes is not well understood. We aimed to compare long-term, self-reported psychosocial and functional outcomes among adult burn survivors who were obese, normal weight, and underweight at discharge.

**Methods:**

Adult burn survivors participating in a national longitudinal, multicenter patient-reported outcomes database between 2015-2023 were included. Participants were divided into three groups: those who were obese (BMI ≥ 30), normal weight (BMI 18.5–29.9), and underweight (BMI < 18.5) at discharge. Survey responses using the PROMIS scales (Pain, Anxiety, Depression, Fatigue, Ability to Participate in Social Roles, and Physical Function); PTSD (PCL-5); Satisfaction with Life (SWL) scale; Pain Intensity on global scale; and return to work at 6-, 12-, and 24-months post-burn injury were analyzed. Linear mixed-effects models were used to compare self-reported outcomes among the three participant groups, while adjusting for sex, age, total burn surface area burned, and the longitudinal nature of the data.

**Results:**

A total of 920 participants met inclusion criteria, of which 306 were obese, 579 normal weight, and 35 underweight. There were no significant differences across all self-reported outcomes between normal weight and obese participants. However, over the three time points, underweight participants reported significantly worse physical function (-4.2 pts, p = 0.005) and social participation (-3.49 pts, p = 0.041) compared to those with normal BMI, but there were no significant differences for pain intensity, anxiety, depression, fatigue, SWL, return to work, and PTSD symptoms.

**Conclusions:**

These findings indicate that underweight burn survivors may experience significantly worse physical function and social integration post-burn compared to normal weight survivors long-term. Although modern healthcare typically places greater concern on health outcomes of obese patients, our findings suggest that underweight adults cannot be overlooked. Low BMI status should be considered when discussing long-term physical and psychosocial recovery among adults living with burn injuries. This may be critical due to impaired wound healing secondary to malnutrition and worsened self-esteem due to social stigma among underweight participants. Greater efforts should be focused on providing optimal nutrition and monitoring weight changes of burn survivors during hospitalization. Underweight burn survivors may also strongly benefit from rehabilitative therapy and social support resources to optimize physical recovery and return to daily life.

**Applicability of Research to Practice:**

Greater attention should be focused on targeting and improving long-term outcomes for underweight burn patients in their process of recovery.

**Funding for the Study:**

The contents of this abstract were developed under a grant from the National Institute on Disability, Independent Living, and Rehabilitation Research. NIDILRR is a Center within the Administration for Community Living (ACL), Department of Health and Human Services (HHS). The contents of this abstract do not necessarily represent the policy of NIDILRR, ACL, or HHS, and you should not assume endorsement by the Federal Government.

